# Chirality Construction from Preferred π-π Stacks of Achiral Azobenzene Units in Polymer: Chiral Induction, Transfer and Memory

**DOI:** 10.3390/polym10060612

**Published:** 2018-06-04

**Authors:** Tengfei Miao, Lu Yin, Xiaoxiao Cheng, Yin Zhao, Wenjie Hou, Wei Zhang, Xiulin Zhu

**Affiliations:** 1 Suzhou Key Laboratory of Macromolecular Design and Precision Synthesis, Jiangsu Key Laboratory of Advanced Functional Polymer Design and Application, State and Local Joint Engineering Laboratory for Novel Functional Polymeric Materials, College of Chemistry, Chemical Engineering and Materials Science, Soochow University, Suzhou 215123, China; mtf0824@163.com (T.M.); yinlu0701@163.com (L.Y.); studentscheng@163.com (X.C.); zhaoyinsuda@126.com (Y.Z.); xlzhu@suda.edu.cn (X.Z.); 2 Global Institute of Software Technology, No 5. Qingshan Road, Suzhou National Hi-Tech District, Suzhou 215163, China

**Keywords:** supramolecular chirality, Azo-containing polymers, chiral aggregation, chiral film

## Abstract

The induction of supramolecular chirality from achiral polymers has been widely investigated in composite systems consisting of a chiral guest, achiral host, and solvents. To further study and understand the process of chirality transfer from a chiral solvent or chiral molecules to an achiral polymer backbone or side-chain units, an alternative is to reduce the components in the supramolecular assembled systems. Herein, achiral side-chain azobenzene (Azo)-containing polymers, poly(6-[4-(4-methoxyphenylazo) phenoxy] hexyl methacrylate) (PAzoMA), with different *M*_n_s, were synthesized by atom transfer radical polymerization (ATRP). Preferred chirality from supramolecular assembled *trans*-Azo units of PAzoMAs is successfully induced solely by the neat limonene. These aggregates of the polymers in limonene solution were characterized by circular dichroism (CD), UV-vis spectra, and dynamic light scattering (DLS) under different temperatures. The temperature plays an important role in the course of chiral induction. Meanwhile, supramolecular chirality can be constructed in the solid films of the achiral side-chain Azo-containing polymers that were triggered by limonene vapors. Also, it can be erased after heated above the glass transition temperature (*T*_g_) of the polymer, and recovered after cooling down in the limonene vapors. A chiroptical switch can be built by alternately changing the temperature. The solid films show good chiral memory behaviors. The current results will facilitate studying the mechanism of chirality transfer induced by chiral solvent and improve potential application possibilities in chiral film materials.

## 1. Introduction

Chirality is a common phenomenon in nature and organisms, such as spiral amino acids, vines, conches, and even the galactic system [1]. The study of chirality in terms of the elemental distribution and molecular structure has always attracted the curiosity of scientists [2]. At the supramolecular level, supramolecular chirality refers to chirality generated by non-covalent interaction between building units, such as hydrogen bonding [3], π-π stacking [4,5], electrostatic interaction [6], and host–guest interaction [7]. The production of supramolecular chirality from achiral polymer building blocks has very important significance in theoretical research and practical application. Many strategies, such as chiral liquid-crystal field [8], chiral solvent [9,10,11], circularly polarized light (CPL) [12,13,14], interfacial interaction [15], and gelation [16] have been reported to produce supramolecular chirality from achiral polymer systems.

The chiral induction of achiral substances by chiral solvent is a flexible and effective method, which is suitable for organic small molecules [17], oligomers [18], and polymer systems [19,20]. The first example of a chiral-solvation-induced chirality of an achiral polymer was demonstrated by Green et al. [21]. The dynamically interconverting helical senses can be found in the circular dichroism (CD) spectrum when dissolving achiral polyisocyanate in several chiral chlorinated solvents. Motivated by this pioneering study, optically active polymers or polymer aggregates, such as polysilanes [22], polyfluorenes [23,24,25], polyacetylenes [26], and azobenzene (Azo)-containing polymers [9,27] have been shown to achieve chirality in aggregation states induced by chiral solvation. Azo-containing polymers are very promising materials in the field of photoswitchable molecular systems and liquid crystalline materials. Due to the dramatic changes in the polarity, shape, and size of the Azo units during the process of photoisomerization, Azo-containing polymers showed unique behaviors of chiroptical switching, which was observed by UV-vis and circular dichroism (CD) spectra [28]. In our group, we have introduced Azo groups into the main chain [9] and side-chain [27] of the achiral polymers, and successively achieved the supramolecular chirality from the achiral building blocks induced by chiral limonene. However, this induction and assembly process of main chain Azo-containing polymers requires a complex system consisting of the good solvent, a chiral solvent, and a weak solvent. Recently, we further simplified the assembled components of a side-chain Azo-containing polymer by using limonene as the weak and chiral solvent simultaneously [27,29,30]. On the one hand, although much effort has been paid to simplifying the assembled components and studying the inducing mechanism, we still have a problem remaining to be solved of whether the construction of supramolecular chirality in the achiral side-chain Azo-containing polymer can be achieved by pure limonene under the controlled temperature. The neat chiral solvent will avoid the interactions between the other solvents (good and poor) and building blocks. 

On the other hand, the chirality transfer and amplification phenomena have been generally observed in polymer solution [22,31,32]. However, the chiral transfer, chiral switching, and memory of supramolecular chirality in solid polymer films are more promising in practical applications, such as chiral nonlinear optics and data storage. Guerra et al. [33] presented that the chiral *s*-PS (syndiotactic polystyrene) films can be obtained through inducing by chiral limonene molecules and then replacing them with their achiral counterparts. The use of circularly polarized light (CPL) has been demonstrated as a method for the chiral induction [34] and chiral regulation [13] of polymer films. Wu et al. reported that the photoinduced circular dichroism of polymer liquid crystals on the thin films can be erased by heating the films above the clearing temperature or by annealing the films in the liquid-crystalline phase [35]. However, the chiral induction of side-chain Azo-containing polymer films by chiral solvent vapors and the modulation of chirality by variable temperature was rarely reported.

In this work, we present the construction of supramolecular chirality from the aggregation of an achiral side-chain Azo-containing polymer in the solution state induced by pure limonene, and in the solid films triggered by chiral limonene vapor. Through a heating-cooling treatment of the polymer solution, the chiral aggregation of achiral Azo-containing polymers can be effectively generated. In the solid film, the chiral limonene vapors can also induce well-organized stacks of Azo units, forming the supramolecular chirality. Meanwhile, the CD signal of the polymer films can be readily erased after heating above the glass transition temperature (*T*_g_) of the polymer, and recovered after cooling it down in the chiral solvent vapors. This regular pattern of chiroptical switch can be repeated effectively at least five times. Besides, the supramolecular chirality of the polymer film can be memorized well.

## 2. Experiment

### 2.1. Materials

1-Chloro-6-hydroxyhexane (Acros, 95%), (*R*)-(+)-limonene (1*R*, TCI, >95%, ${\left[\alpha \right]}_{589}^{24}$ = +99.62°, TCI Shanghai Development Co., Ltd., Shanghai, China) and (*S*)-(−)-limonene (1*S*, TCI, >95%, ${\left[\alpha \right]}_{589}^{24}$ = −97.72°, TCI Shanghai Development Co., Ltd., Shanghai, China) were used without further purification. Copper (I) bromide (CuBr, Aldrich, St. Louis, MO, United States, 98%) was purified with glacial acetic acid and washed with pure ethanol, and then stored under argon before use. Ethyl 2-bromoisobutyrate (EBiB) (TCI, 98%), *N,N,N′,N,′N″*-pentamethyldiethylenetriamine (PMDETA) (Aldrich, St. Louis, MO, United States, 97%), and the monomer, 6-[4-(4-methoxyphenylazo) phenoxy]hexyl methacrylate (AzoMA_6_) were synthesized as described previously [36], which was confirmed by the ^1^H and ^13^C NMR data (Figure S1). ^1^H NMR (CDCl_3_, 300 MHz), (δ, ppm): 7.87 (m, 4H), 6.99 (m, 2H), 7.26 (m,2H), 6.10 (s, 1H), 5.54 (s, 1H), 4.18 (t, 2H), 4.03 (t, 2H), 3.92 (s, 3H), 1.98 (s, 3H), 1.81 (m, 2H), 1.71 (m, 2H), 1.50 (m, 4H). Elemental analysis, calculated values for C_23_H_28_N_2_O_4_: C, 69.68; H, 7.12; N, 7.07. Found: C, 69.32; H, 6.95; N, 7.11.

### 2.2. Synthesis of Poly(6-[4-(4-methoxyphenylazo) phenoxy] Hexyl Methacrylate (PAzoMA)

AzoMA (0.5 g, 1.26 mmol), EBiB (12.3 mg, 0.063 mmol), PMDETA (10.94 mg, 0.063 mmol), CuBr (9.05 mg, 0.063 mmol), and THF (1.5 mL) were added to a 5-mL ampoule. Then, the ampoule was flame-sealed after being deoxygenated with three standard freeze—pump—thaw cycles. The polymerization under argon atmosphere was carried out at 70 °C for 1.5 h. The mixture was diluted with 1 mL of THF, passing a column of neutral Al_2_O_3_, and then precipitated into an excess of methanol (50 mL) twice. After collection by filtration, the polymer was dried in a vacuum oven overnight at 30 °C (0.348 g, 69.5%). Another polymer with different *M*_n_ was prepared by adjusting the molar ratio of the monomer and initiator with the similar procedures. The molecular weights and molecular weight distributions of the obtained two polymers are listed in Table 1.

### 2.3. Preparation of the Optically Active Polymer Aggregates in Solution

A small amount of polymer solid (0.1 mg) was added to 3 mL of (*R*)-(+)-limonene (1*R*) in a quartz cell. The suspension was stirred under 100 °C for 1 h to make sure the polymer was dissolved completely. The concentration of polymer repeating units is 8.42 × 10^−5^ mol L^−1^. The optically active polymer aggregates were prepared after the solution was cooled down to room temperature. The other polymer aggregates were prepared in a similar way. This yellowish turbid solution of PAzoMA aggregates was employed for CD/UV-vis measurements.

### 2.4. Preparation of the Polymer Solid Films

The 10 mg/mL polymer solution was obtained by solving 10 mg of polymer solid in 1 mL of CHCl_3_. A thin film sample of PAzoMA was prepared by spin coating about 0.1 mL of the polymer solution onto a clean quartz plate at a speed of 0.5 rpm for 6 s and a speed of 1.9 rpm for 20 s. After being dried and annealed under vacuum at 90 °C for 12 h to drive off residual solvent, the film was stored in darkness for further study.

### 2.5. Chiral Induction Process of the Films

The films obtained by the above method were measured with UV-vis and CD spectra. First, the film was fixed in the cell, and suspended above the surface of limonene (0.1 mL). Then, the vapors of limonene would be produced with the temperature increasing, and the optically active polymer film was achieved at the same time. The results of chiral assembly were recorded by UV-vis and CD spectra when the temperature was alternately changed between 70 °C and 60 °C. 

### 2.6. Characterization

Gel permeation chromatograph (GPC) measurements were conducted on the TOSOH HLC-8320 gel permeation chromatogragh (GPC) (Tokyo, Japan), which was equipped with a refractive index and UV detectors using two TSKgel SuperMultiporeHZ-N (4.6 × 150 mm, 3.0 μm beads size) columns (Tokyo, Japan) arranged in a series. It can separate polymers in the molecular weight range of 500–190k Da. THF was used as the eluent with a flow rate of 0.35 mL/min at 40 °C. The values of the average molecular weight (*M*_n_) and molecular weight distribution (*M*_w_/*M*_n_) of the samples were calculated with polymethyl methacrylate (PMMA) standards. ^1^H NMR spectra of the polymers were recorded on a Bruker nuclear magnetic resonance instrument (300 MHz, Brucker, Kalsruhe, Germany) using CDCl_3_ as the solvent and tetramethylsilane (TMS) as the internal standard at 25 °C. The UV-vis spectra were recorded on a UV-2600 spectrophotometer (Shimadzu (Nakagyo-ku, Kyoto, Japan)). The CD spectra were recorded on a JASCO J-815 spectropolarimeter equipped with a Peltier-controlled housing unit using a SQ-grade cuvette, a single accumulation, a path length of 10 mm, a bandwidth of 2 nm, a scanning rate of 200 nm min^−1^, and a response time of 1 s. The samples were measured at different temperatures. The magnitude of the circular polarization at the ground state was defined as *g*_CD_ = 2 × (*ε*_L_ − *ε*_R_)/(*ε*_L_ + *ε*_R_), where *ε*_L_ and *ε*_R_ denoted the extinction coefficients for left and right circularly polarized light, respectively. Experimentally, the *g*_CD_ value was defined as Δ*ε*/*ε* = [ellipticity/32,980]/absorbance at the CD extremum. Elemental analyses (C, H, and N) were measured with an EA1110 CHNO-S instrument. The thermal behavior and glass transition temperatures of PAzoMA were measured using a TA-Q100 DSC instrument (New Castle, DE, USA). Dynamic light scattering (DLS) measurements were performed with a Zetasizer Nano ZS instrument (Brookhaven, Holtsville, TX, USA) at different temperatures. 

## 3. Results and Discussion

### 3.1. Synthesis and Characterization of Side-Chain Azo-Containing Polymers

**Scheme 1:**
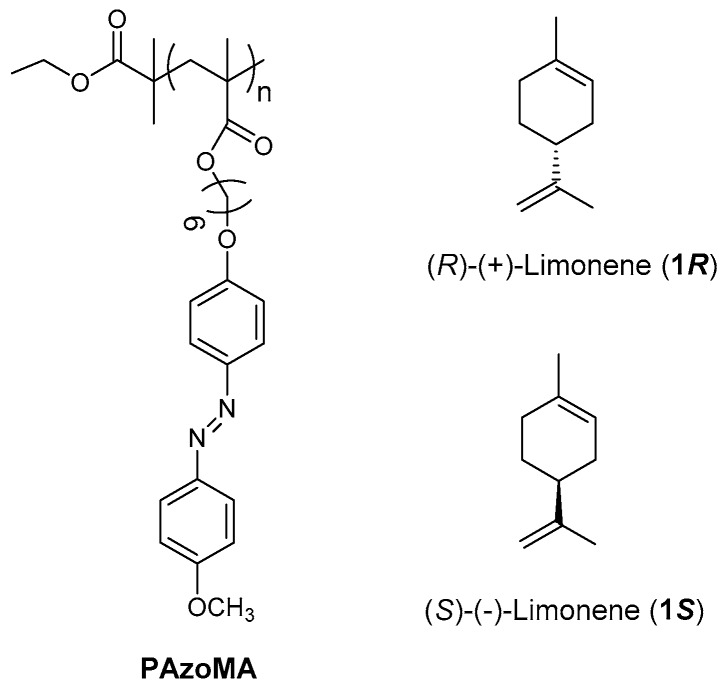
Illustration of the chemical structures of poly(6-[4-(4-methoxyphenylazo) phenoxy] hexyl methacrylates) (PAzoMAs), (*R*)-(+)-limonene (1*R*) and (*S*)-(−)-limonene (1*S*).

The homopolymers of AzoMA (PAzoMA) (Scheme 1) were prepared by atom transfer radical polymerization (ATRP) [36] with a controlled molecular weight (*M*_n_) and relatively low molecular weight distribution (*M*_w_/*M*_n_) (Figure S2). As presented in Table 1, the side-chain Azo-containing polymers with different *M*_n_s (7400 g mol^−1^ and 12,400 g mol^−1^) and relative low molecular weight distribution (*M*_w_/*M*_n_) (1.14 and 1.20) were successfully prepared by changing the molar ratio of the monomer and initiator during the polymerization process.

### 3.2. The Chiral Aggregation of the Azo-Containing Polymer in Neat Limonene Solution 

As was previously reported, typically a good solvent, chiral solvent, and poor solvent are required to produce supramolecular chiral aggregation from achiral conjugated polymers [27,29]. Indeed, the chiral-solvation-induced chirality by single solvent can greatly simplify the research system. In this work, the limonene (1*R* and 1*S* in Scheme 1) was simultaneously chosen as the chiral solvent, poor solvent, and good solvent. The polymer solid can be dissolved in limonene completely after stirring for 1 h at relatively high temperature (100 °C). Then, the supramolecular aggregation of the polymer occurred after cooling down the solution. The temperature is a very important factor in the supramolecular assembly of Azo-containing polymers. When the temperature is higher than 70 °C, the well-dissolved polymer chains in limonene may result in a relatively weak π-π stacking of Azo units in polymer side chains. The optically active polymer aggregates would be produced due to a stronger π–π stacking of Azo units after the temperature drops below 70 °C. This result can be revealed by DLS, UV-vis, and CD spectra.

**Figure 1:**
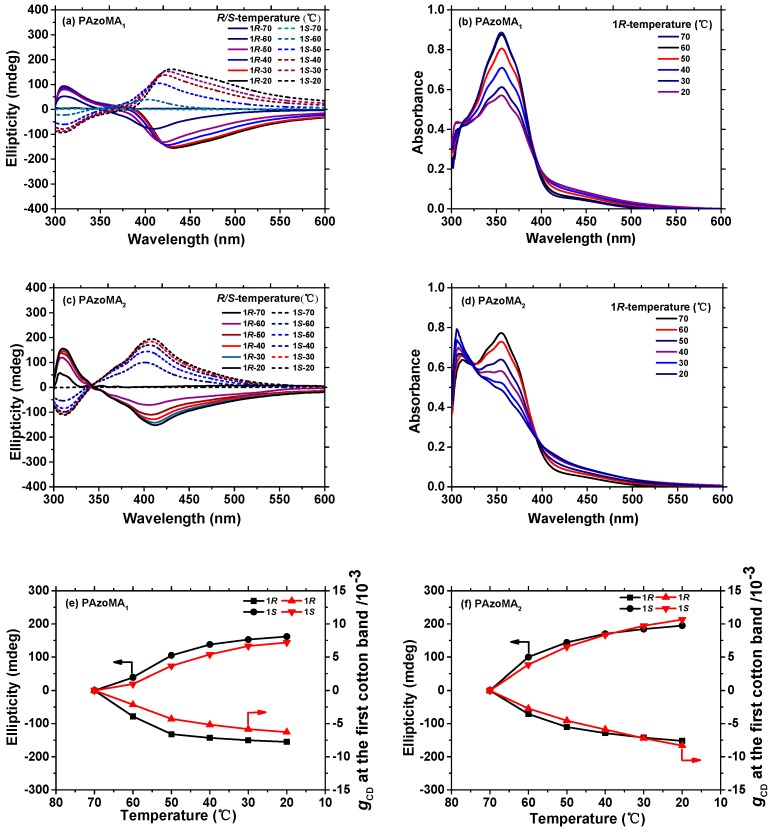
The circular dichroism (CD) (**a**,**c**), UV-vis spectra (**b**,**d**) and maximum CD and *g*_CD_ values (**e**,**f**) of the side-chain Azo-containing polymer aggregates with the temperature decreasing from 70 °C to 20 °C. (**a**,**b**,**e**) stand for PAzoMA_1_, (**c**,**d**,**f**) stand for PAzoMA_2_. The concentration of polymer repeating units is 8.42 × 10^−5^ mol L^−1^.

For the side-chain Azo-containing polymers (PAzoMA_1_ and PAzoMA_2_), the UV-vis spectra (Figure 1b,d and Figure S3) of the polymer aggregates in limonene (1*R* or 1*S*) consists of two absorption bands. The absorption bands ranging from 320 nm to 400 nm are attributed to the π-π* electronic transition of the *trans*-Azo, and the others from 400 nm to 500 nm are attributed to the n—π* electronic transition of the *cis*-Azo. With the temperature decreasing, absorption of the π-π* band became lower and wider, indicating that the strong π-π stacking of Azo units in the polymer side chain occurred.

The intense and mirror-image cotton effects can be found in the CD spectrum. The CD signals are related to the π-π* electronic transition of the *trans*-Azo, demonstrating that the supramolecular chirality could be successfully introduced to the side-chain of the Azo-containing polymers by the neat chiral solvent (limonene). As shown in Figure 1a,c,e,f, with the temperature decreasing from 70 °C to 20 °C, the CD and *g*_CD_ values of the aggregates began to gradually increase. The relatively poor solubility of the polymers in limonene increased the degree of aggregation, which resulted in the increasing intensity of the CD signals. These results confirm our conjecture. Compared with the chiral assembly in the mixed solvents [27], the maximum CD and *g*_CD_ values of the aggregates in neat limonene are much higher. Furthermore, PAzoMA_2_ with a high molecular weight gives higher CD and *g*_CD_ absolute maximum values (Figure 1e,f), resulting from the much higher degree of chiral aggregation. The stronger chiral aggregation behavior of PAzoMA_2_ compared with PAzoMA_1_ can be observed in their UV-vis spectra (Figure 1b,d). The relatively lower UV-vis absorption intensity and wide spectra demonstrated the stronger aggregation by π-π stacking of Azo units in the polymer side chain. 

**Figure 2:**
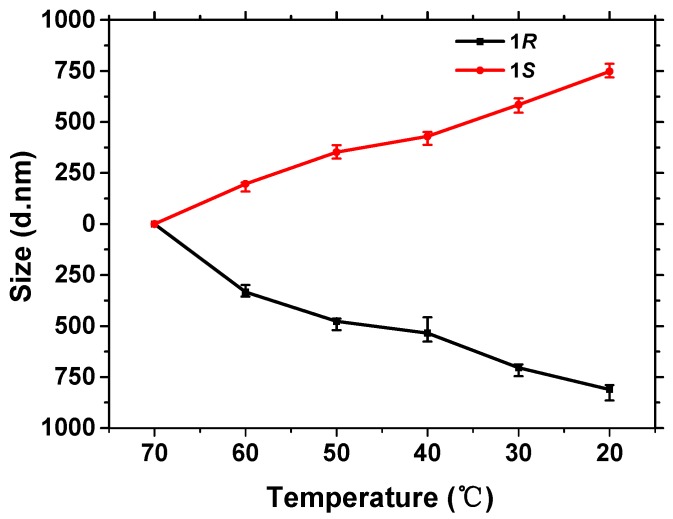
The dependence of the PAzoMA_1_ aggregates’ size on the temperature, which is decreasing from 70 °C to 20 °C. These date are taken from Figure S4a,b. The concentration of polymer repeating units is the same as in Figure 1.

The above results can also be proved by dynamic light scattering (DLS). With the temperature decreasing from 70 °C to 20 °C, the size of the polymer aggregates becomes bigger, ranging from 0 nm to 810 nm in *R*-limonene, and from 0 nm to 784 nm in *S*-limonene (Figure 2). 

### 3.3. Supramolecular Chirality of Polymer Solid Films

**Figure 3:**
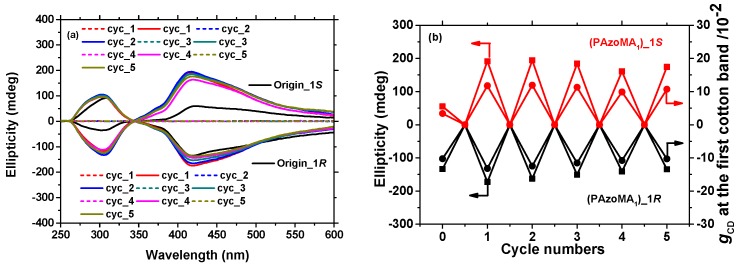
Changes in CD spectra (**a**) and the maximum CD and *g*_CD_ values (**b**) of PAzoMA_1_ films during heating to 70 °C and cooling to 60 °C five times.

The liquid-crystalline phase transition of side-chain Azo-containing polymers has been intensively investigated in many groups [37,38,39]. According to the DSC results (Figure S5), the glass transition temperature is around 65 °C for both PAzoMA_1_ and PAzoMA_2_. In the case of a polymer solid film, a small amount of 1*R* or 1*S* limonene (0.1 mL) was added to the quartz cell. After that, the polymer solid film was fixed in the cell and suspended above the limonene surface. When 1*R* was added, the intense negative CD signal related to the π-π* electronic transition of the *trans*-Azo chromophore was observed at 60 °C, indicating that the optically active polymer film was successfully produced by limonene vapor. The absorption of the π-π* band began to decrease in the UV-vis spectra (Figure S6) when the temperature reached 70 °C beyond *T*_g_. The chiral signal in the CD spectra disappeared immediately at the same time. The reason may be that the chain segment of the polymer would begin to move randomly after heating above its *T*_g_ temperature, which would destroy the well-organized helical π-π stacking of the Azo units induced by limonene vapor. Similar results were found in the thin films of achiral liquid crystal polymers induced by CPL [35]. Interestingly, when the polymer films were cooled to 60 °C again, the polymer chains cooled down in the environment of the chiral vapors, and the chiral aggregates could re-form again. Chiral signals similar to the previous intensity were observed in the CD spectrum. We successfully built a chiral switch of the polymer solid film by alternately changing the temperature between 70 °C and 60 °C. Five switching cycles were tested, and the obvious decline of the maximum absolute CD amplitude was not found, as presented in Figure 3. Almost intense mirror-image CD spectra were obtained when 1*R* was replaced by 1*S*. 

### 3.4. Chiral Memory Property of Azo-Containing Polymer Film

**Figure 4:**
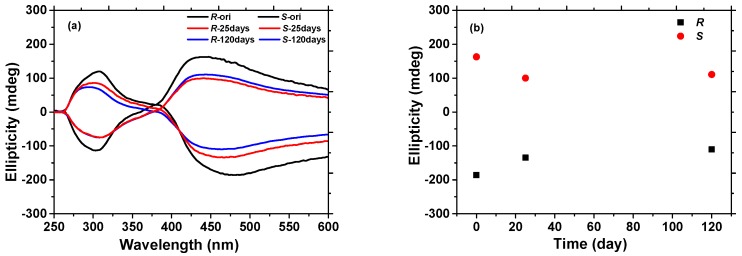
Changes in the CD spectra (**a**) and the maximum CD (**b**) of the PAzoMA_1_ aggregates in the films without the existence of limonene.

Due to the strong molecular interaction occurring in the polymer chains in the solid state, the polymer films always present a superior chiral memory performance [40]. In this case, the supramolecular chirality was firstly induced in thin films of the achiral side chain Azo-containing polymers by limonene vapors. After that, the residual limonene molecular was removed at 40 °C under vacuum for 24 h, and then stored in a fume hood for several months. The content of limonene was measured by ^1^H NMR and described in Figure S7. There would be a slight attenuation of the CD signals in the PAzoMA films at the beginning, and then, the signals could maintain a stable intensity for several months. The results can be proved in Figure 4.

### 3.5. Chiral Amplification

**Figure 5:**
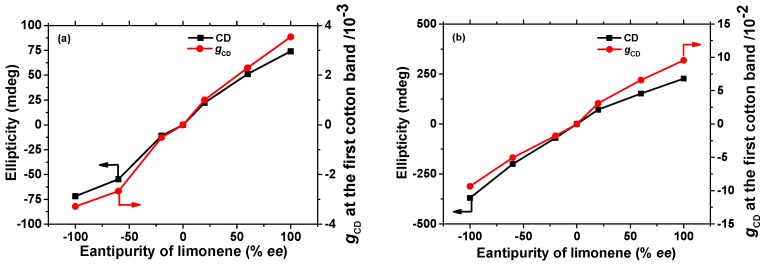
Plots of maximum CD and *g*_CD_ values of the PAzoMA_1_ in the solution (**a**) and films (**b**) with different enantiopurity of limonene. These data were taken from Figure S8. The solution and films were both heated to 70 °C and measured after being cooled down to 60 °C.

By varying the enantiomeric excess (*ee*) of chiral limonene, the possibility of chiral amplification in the aggregation of Azo units were investigated. While keeping the temperature of the system as a constant (60 °C), the linear plot of maximum CD and *g*_CD_ values (Figure 5) demonstrated that the chiral aggregation of Azo units was linearly controlled by the respective molar ratio of enantiomers (1*R*/1*S*). No obvious chiral amplification occurred while changing the enantiomeric excess (*ee*) of the chiral solvent. A similar result was also found in the polymer solid films. The chiral signals of the aggregates in the thin films can be adjusted by changing the *ee* of the chiral limonene vapors. Similar results were also reported in the limonene-induced supramolecular chirality of π-conjugated main-chain polymers [9,40] and side-chain polymers [27,29]. 

## 4. Conclusions

In summary, the supramolecular chirality was successfully introduced to achiral side chain Azo-containing polymers by heating-cooling treatment in pure limonene solution. With the decrease of temperature, the chiral signals of aggregates tended to increase. Besides, the supramolecular chirality of the polymer solid films could be induced by limonene vapors. The chiral supramolecular structure can be destroyed by heating the film above the glass transition temperature (*T*_g_) of the polymer due to the irregular movements of the polymer chains. Meanwhile, it can be recovered by cooling down the film in the environment of limonene vapors. This reversible chiral—achiral switching process can be repeated more than five times. Furthermore, the supramolecular chirality can be perfectly memorized in the solid state. We successfully achieved the supramolecular chirality of the achiral side chain Azo-containing polymers in the neat chiral limonene and the thin films induced by chiral limonene vapor.

## Figures and Tables

**Table 1:** Molecular weight characteristics of the Azo-containing polymers.

Entry	Ratio ^a^	Conv. ^b^ (%)	*M*_n(th)_^c^ (g mol^−1^)	*M*_n(GPC)_^d^ (g mol^−1^)	*M*_w_/*M*_n_^d^
PAzoMA_1_	20:1:1:1	69.5	5500	7400	1.20
PAzoMA_2_	70:1:1:1	53.4	14,800	12,400	1.14

^a^ Polymerization ratio: [AzoMA]_0_/[EBiB]_0_/[CuBr]_0_/[pentamethyldiethylenetriamine (PMDETA)]_0_. ^b^ Determined gravimetrically. ^c^ Calculated by *M*_n(th)_ = ([Monomer]_0_/[EBiB]_0_) × *M*_w,monomer_ × Conversion%. ^d^ Determined by gel permeation chromatograph (GPC) according to polymethyl methacrylate(PMMA) standards in THF.

## Supplementary Materials

The following are available online at http://www.mdpi.com/2073-4360/10/6/612/s1. Figure S1: ^1^H NMR (a) and ^13^C NMR (b) spectra of AzoMA, Figure S2: GPC curves of PAzoMAs, Figure S3: UV-vis spectra of Azo-containing polymer aggregates in 1*S* with the temperature decreasing from 70 °C to 20 °C. (a) stands for PAzoMA_1_ and (b) stands for PAzoMA_2_, Figure S4: The dependence of PAzoMA_1_ aggregates size in limonene under different temperature, Figure S5: DSC heating curves of PAzoMA_1_ and PAzoMA_2_, Figure S6: UV-vis spectra of the polymer films under limonene vapors during the process of changing the temperature, Figure S7: ^1^H NMR spectra of the residual limonene on the polymer films after chiral induction (a) and placed in the fume hood for 45 days (b), Figure S8: Changes in CD and UV-vis spectra of Azo polymer (PAzoMA_1_) aggregates in the solution (a) and films (b) with different enantiopurity of limonene.
