# Supplementary material for: Chirality Construction from Preferred π-π Stacks of Achiral Azobenzene Units in Polymer: Chiral Induction, Transfer and Memory
Source: Polymers (Basel). 2018 Jun 4;10(6):612. doi: 10.3390/polym10060612 (PMC6404070; doi:10.3390/polym10060612)
Supplement: Supplementary file 1 [file polymers-10-00612-s001.pdf]

Supplementary Materials

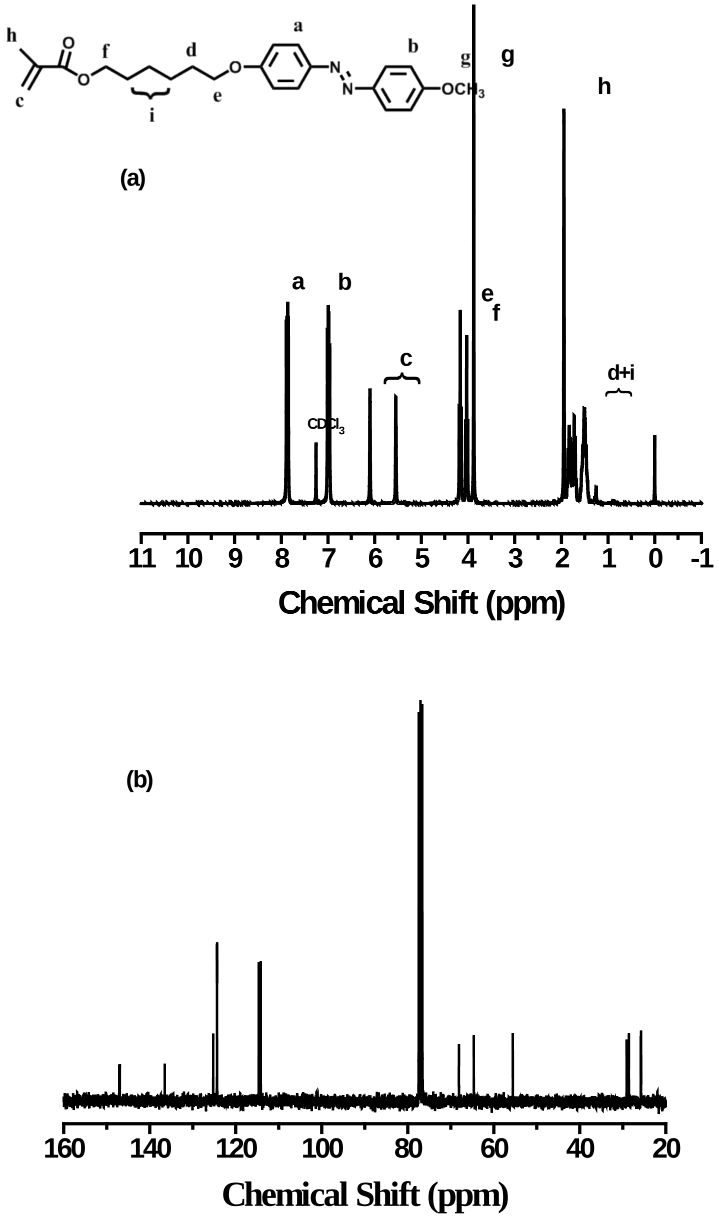

Figure S1. ^{1}H NMR (a) and ^{13}C NMR (b) spectra of the Azo monomer (AzoMA)$_6$.

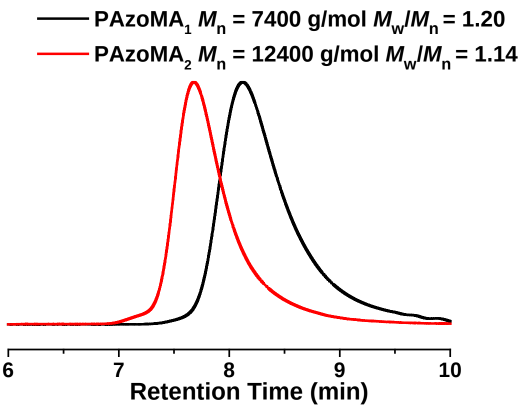

Figure S2. GPC curves of side-chain Azo-containing polymers (PAzoMAs) with different M_ns.

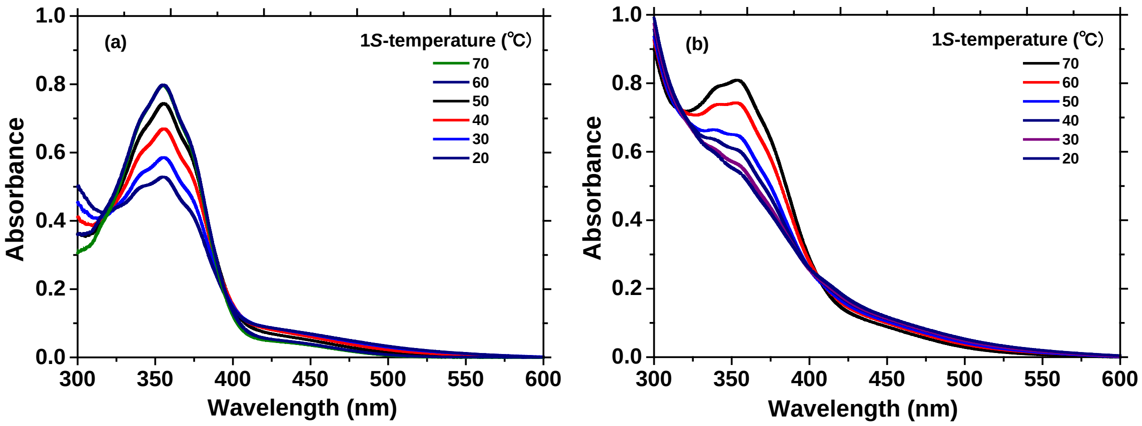

Figure S3. UV-vis spectra of Azo-containing polymer aggregates in 1*S* with the temperature decreasing from 70 °C to 20 °C. (a) stands for $PAzoMA_1$ and (b) stands for $PAzoMA_2$. The concentration of polymer repeating units is 8.42×10^{-5} mol L^{-1}.

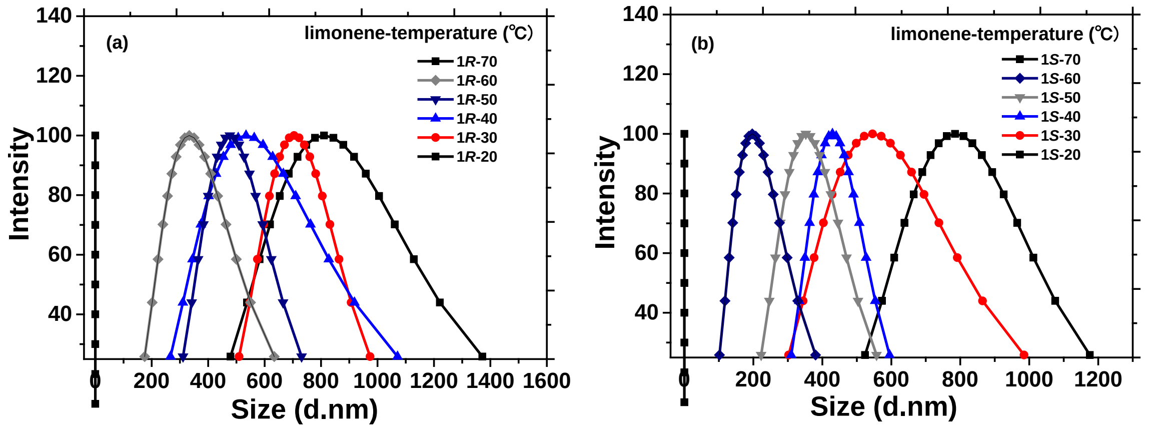

Figure S4. The dependence of PAzoMA1 aggregates size in limonene on the temperature. The concentration of polymer repeating units is the same as in Figure S3.

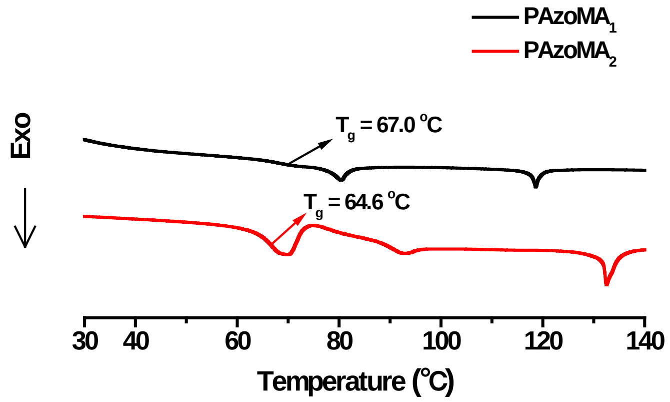

Figure S5. DSC heating curves of PAzoMA1 and PAzoMA2.

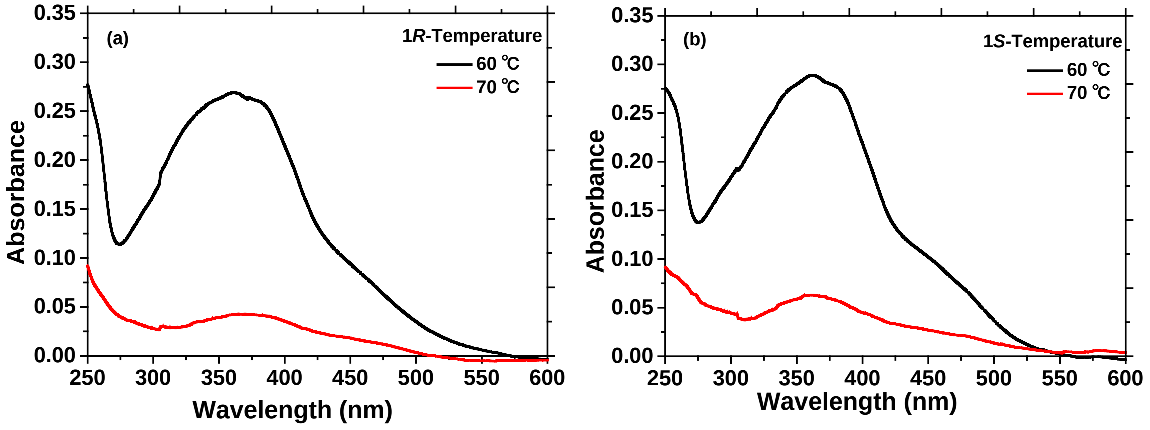

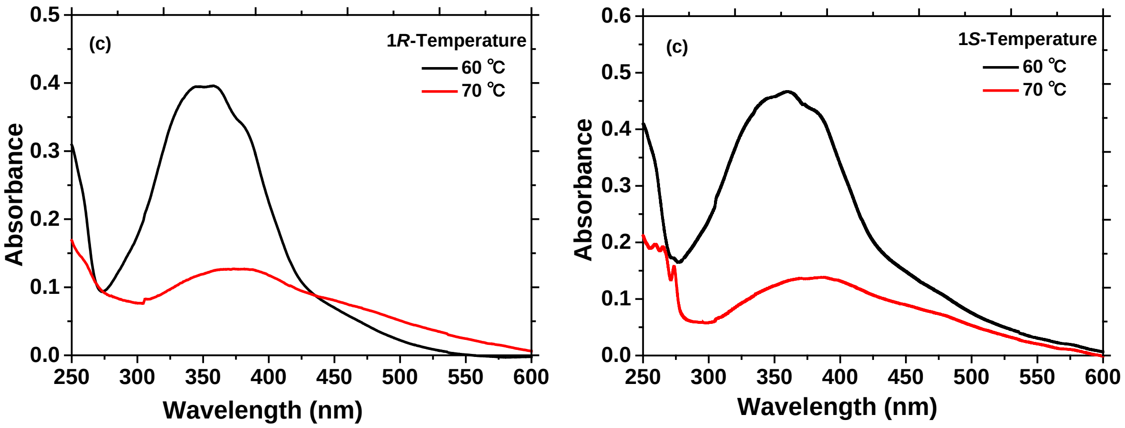

Figure S6. UV-vis spectra of the polymer films under limonene vapors during the process of changing the temperature. 1a and 1b stand for PAzoMA$_1$ films under 1*R* and 1*S*, 2a and 2b stand for PAzoMA$_2$ films under 1*R* and 1*S*, respectively.

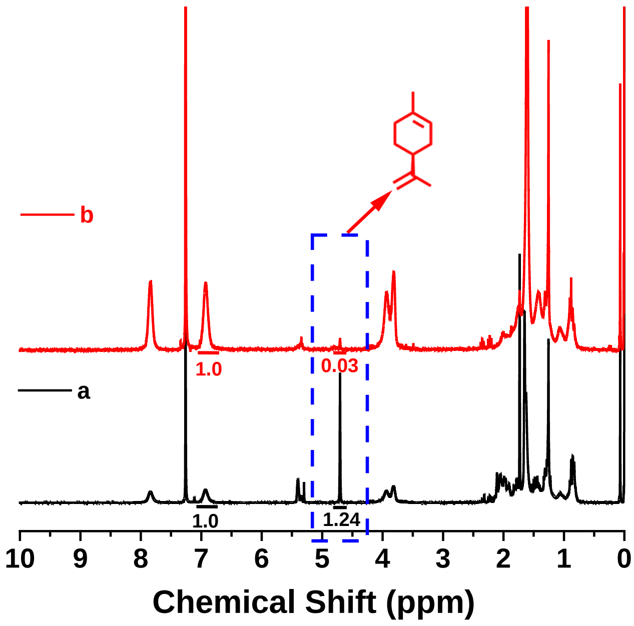

Figure S7. ^{1}H NMR spectra of the residual limonene on the polymer films after chiral induction (a) and (b) placed in the fume hood for 45 days.

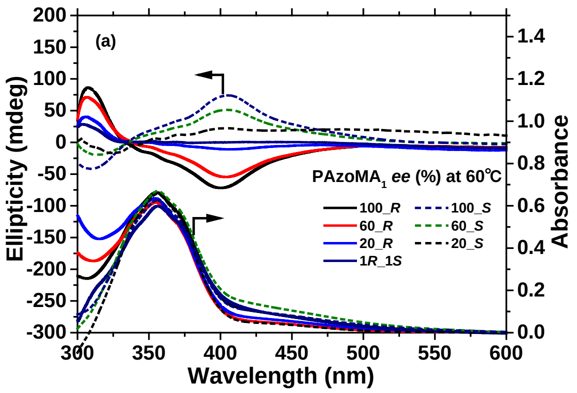

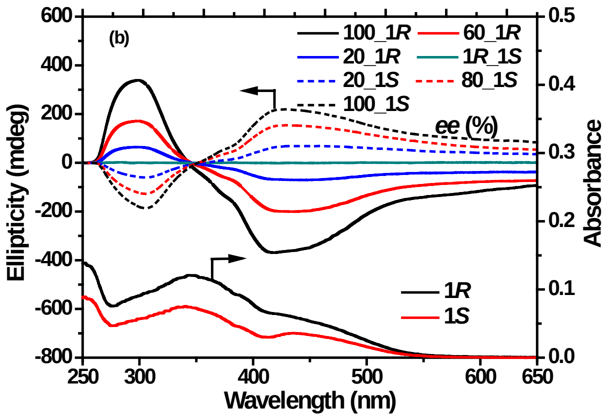

Figure S8. Changes in CD and UV-vis spectra of Azo polymer (PAzoMA$_1$) aggregates in the solution (a) and films (b) with different enantiopurity of limonene. The solution and film were both heated to 70 °C and measured after cooled down to 60 °C.
